# Primary intracranial neuroendocrine tumor: two case reports

**DOI:** 10.1186/s12957-016-0887-4

**Published:** 2016-04-30

**Authors:** Hailong Liu, Haoran Wang, Xueling Qi, Chunjiang Yu

**Affiliations:** Department of Neurosurgery, Sanbo Brain Hospital Capital Medical University, No. 50 Xiang Shan Yi-Ke-Song, Haidian District, Beijing, 100093 People’s Republic of China

**Keywords:** Neuroendocrine tumor, Ectopic ACTH syndrome, Anterior skull base reconstruction, Diagnosis, Treatment

## Abstract

**Background:**

Neuroendocrine tumor originates from the diffuse neuroendocrine system. Intracranial originating is lower to 0.74 %.

**Case presentation:**

We present two cases of primary intracranial neuroendocrine tumor A 39-year-old woman was admitted with headache, fever, polydipsia and polyuria. Biochemical and endocrinological results showed hyponatremia, hypothyroidism and hypopituitarism. MRI scans demonstrated an obviouslyenhancing lesion in seller and superseller area. Then a gross removal of tumor was achieved during the single nostril transsphenoidal approach surgery. Pathological diagnosis was high-grade small-cell neuroendocrine tumor. A 40-year-old woman presented with multiple symptoms and neurological deficit. Neuroimaging results demonstrated a huge obviously-enhancing tumor in anterior cranial fossa. Biochemical and hormone findings revealed hypokalemia, high glucose and hypercortisolemia. The intracranial surgery achieved a gross removal through a right frontal craniotomy. Pathological diagnosis was low-grade small-cell neuroendocrine tumor with immuno-negativity for ACTH.

**Conclusion:**

The mechanism, diagnosis, and treatment of neuroendocrine tumor are still challenging.

## Background

Neuroendocrine tumor originates from the diffuse neuroendocrine system. Intracranial origin is lower than 0.74 %. Ectopic secretion of active adrenocorticotropic hormone (ACTH) or ACTH analogues from the non-pituitary tumors causes ectopic ACTH syndrome, resulting in the presence of Cushing syndrome. Anterior skull base reconstruction was performed during surgery in the second case. Reconstruction can be divided into soft-tissue reconstruction and bony reconstruction. We present two cases of primary intracranial neuroendocrine tumor and the specificities are intracranial origin, immuno-negativity for ACTH, high serum ACTH level, and anterior skull base reconstruction.

## Case presentation

### The first case

**Fig. 1:**
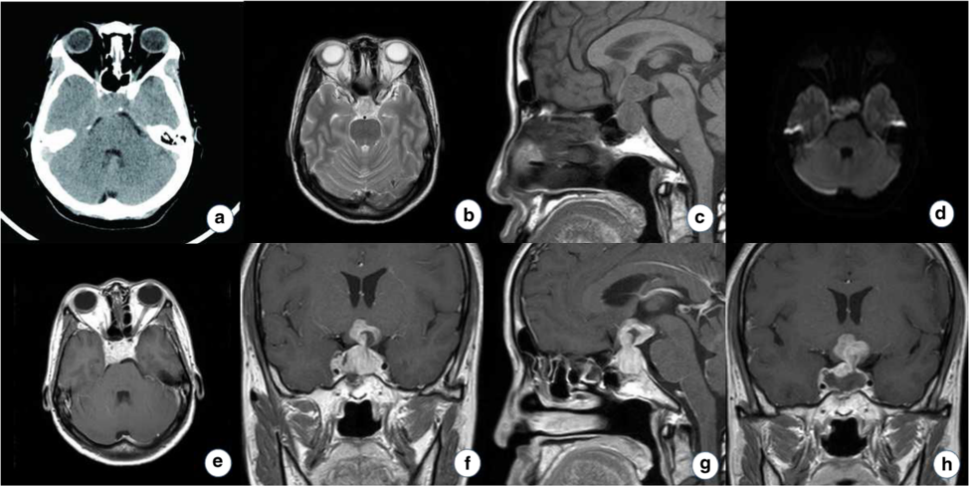
The neuroimaging results of the first patient. **a** CT scans demonstrated an oval high-density lesion in the sellar area and bone destruction. **b** T2-weighted MRI showed the lesion was slight hyperintense. **c** T1-weighted sagittal MRI showed the lesion was isointense. **d** DWI-MRI showed the lesion was iso- or hyperintensity. **e**–**g** The solid part heterogeneously enhanced after contrast. **h**. Postoperative MRI scans (3 days later) showed a gross removal of the tumor

**Fig. 2:**
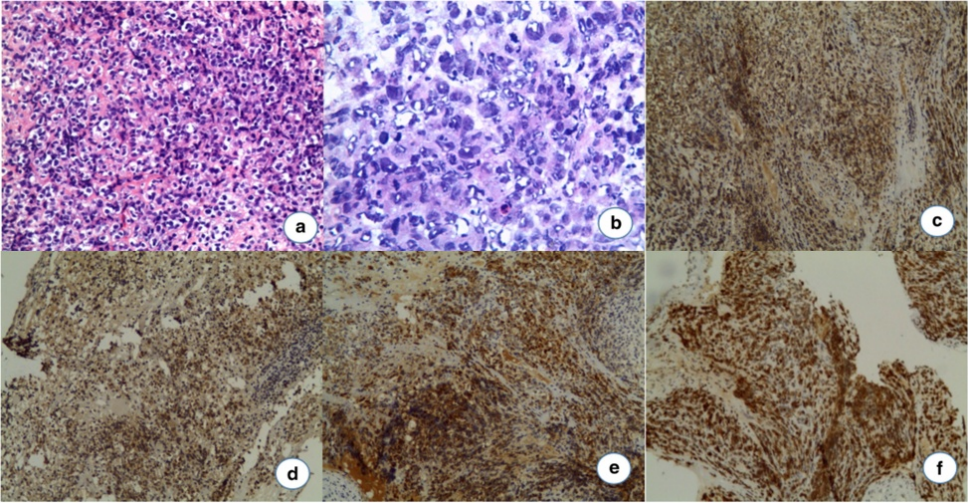
The pathological results of the first patient. **a**, **b** Specimen was comprised of different size cells with distinct atypia and proliferated actively. (HEA 100×, B 200×). **c**–**e** Tumor cells were immunohistochemical positive for Syn (**c**), ChrA (**d**), and CK8 (**e**). **f** The Ki-67-labeling index was more than 90 % in the atypical cells. (IHC 100×)

A 39-year-old woman was admitted to our department with a 2-month history of headache, moderate fever, polydipsia, and polyuria. Biochemical and endocrinological results showed that hyponatremia, mild hypothyroidism, and 6 a.m. serum cortisol level was less than 80.00 nmol/L (83.00~359.00 nmol/L). Bone destruction was found in computed tomography (CT) scans (Fig. 1a). Magnetic resonance imaging (MRI) scans demonstrated an oval obviously enhancing lesion in the sellar and hypothalamic areas with hypointensity on T1-weighted MRI and mixed signals on T2-weighted MRI (Fig. 1b–g). The preoperative diagnosis was sellar and suprasellar neoplasm and hypopituitarism. Then, a gross removal of tumor was achieved via the right single-nostril transsphenoid approach (Fig. 1h). Morphological examination revealed that the tumor was comprised of different size cells with distinct atypia, which were arranged in disperse and sheet form and proliferated actively (Fig. 2a, b). The specimen was immunohistochemical positive for epithelial markers, including CK8, CK18, and cytokeratin A (CgA), and also positive for neuroendocrine markers, including synaptophysin (Syn) and chromogranin A (ChrA), but negative for GFAP, CEA, epithelial membrane antigen (EMA), and thyroid transcription factor-1 (TTF1) (Fig. 2c–e). The Ki-67-labeling index was more than 90 % (Fig. 2f). The pathological diagnosis was high-grade small-cell neuroendocrine tumor (NET). Radiotherapy was recommended to control the growth of the malignancy, but the patient died of extensive metastasis after about 3 months.

### The second case

**Fig. 3:**
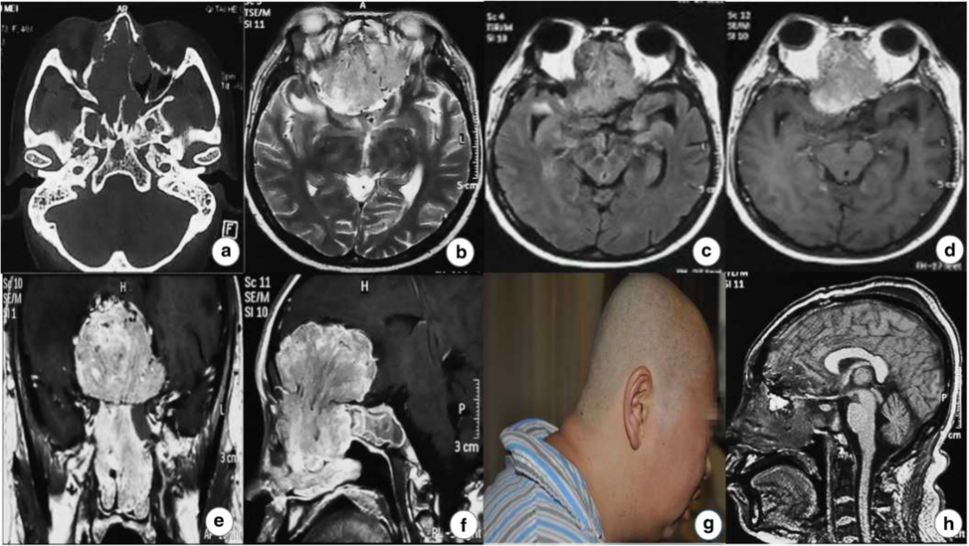
The radiological results and portraiture of the second patient. **a** Bone CT scans showed the neoplasm in the anterior cranial fossa eroded the bilateral sphenoid and the ethmoid sinus bone. **b** T2-weighted MRI showed the lesion was slight hyperintense. **c** The lesion showed hyperintensity in FLAIR MRI. **d**–**f** MRI scans demonstrated the obviously enhancing tumor infiltrated the nasal cavity, and some striped flow-empty action was found on the superior tumor border. **g** Preoperative picture showed the patient with fat centripetal distribution and pigmentation in the nape. **h** Postoperative MRI (1 week later) scans showed a gross removal of the intracranial tumor was achieved, but most part of it in the nasal cavity was removed

A 40-year-old woman was admitted to our institute with a 6-year history of intermittent sniveling from bilateral nostrils; a 2-month history of gained weight, polydipsia, polyuria, and weakness; and a 2-week history of dimness. Examination revealed hypertension, dimness, bilateral exorbitism, left vision diminution, pigmentation in the face and trunk (Fig. 3g). Bone destruction was found in bone CT scan (Fig. 3a). T2-weighted MRI showed the lesion was slight hyperintense, and some spot flow-empty action was found on the superior tumor border (Fig. 3b). The neoplasm in the anterior cranial fossa obviously enhanced after contrast, which infiltrated the sphenoid, ethmoid, and left maxillary sinus as well as the nasal cavity (Fig. 3c–f). The abdomen CT scans showed bilateral adrenal hyperplasia. The serum potassium ranged 1.92~2.82 mmol/L, and the fasting blood glucose (FBG) ranged 14.90~20.00 mmol/L. Hormone checking results showed that the basal ACTH level was 33.72 pmol/L (<2.20 pmol/L), the 6 a.m. serum cortisol level was 1779.78 nmol/L (83.00~359.00 nmol/L), and the testosterone level was 8.43 nmol/L (0.17~2.53 nmol/L). The provisional diagnosis was anterior cranial base and nasal cavity communicating tumor with ectopic ACTH syndrome. The gross removal of the intracranial tumor was achieved, but most part of it in the nasal cavity was removed through a right subfrontal craniotomy (Fig. 3h). Meanwhile, anterior skull base reconstruction was performed during surgery.

**Fig. 4:**
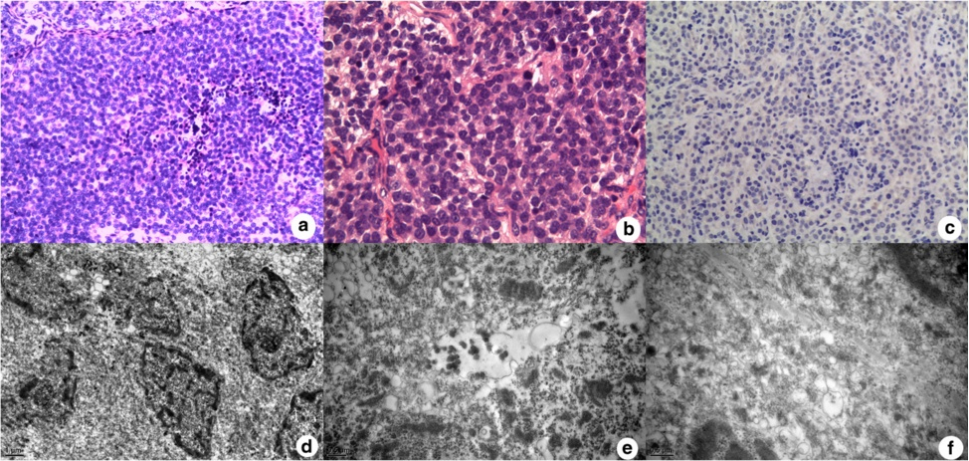
The pathological results of the second patient. **a**, **b** The tumor was comprised of small round cells with uniform nuclei and scant cytoplasm. (HEA 100×, B 400×) **c** The Ki-67-labeling index was less than 1 %. **d**–**f** Electron micrographs showed that neuroendocrine granules (NEGs) were occasionally observed. (D 10,000×, E 20,000×, F 25,000×)

Histological examination revealed that the tumor was comprised of small round cells with uniform nuclei and scant cytoplasm, with nidulant or stripe-shaped distribution (Fig. 4a, b). Immunohistochemically, the cells were positive for epithelial markers, such as CK8, CK18, and CgA, and also positive for neuroendocrine markers, such as Syn, ChrA, and NSE, but negative for CEA, EMA, TTF1, and ACTH. The Ki-67-labeling index was less than 1 % (Fig. 4c). Electron micrographs showed that neuroendocrine granules (NEGs) were occasionally observed (Fig. 4d–f). These examinations suggested that the pathological diagnosis was low-grade small-cell NET.

Postoperative abdomen CT scans demonstrated improved bilateral adrenal glands. Biochemical and endocrine results were normal.

### Discussion

#### Neuroendocrine tumor

NET originates from amine precursor uptaking and decarboxylation cells (APUD cells), which are called diffuse neuroendocrine system [1–3]. the intracranial origin is lower than 0.74 % [4]. To the best of our knowledge, this is the first case of primary intracranial NET with Cushing syndrome which is immuno-negative for ACTH. Malignancy is divided into three levels according to the Ki-67-labeling index, with 1~3 % low level, 3~20 % middle level, and up to 20 % high level. The first patient had a rapid progress because of the high proliferation. The second neoplasm developed slowly because the Ki-67 was lower than 1 %. Patients with NET commonly present with no specific clinical features, including focal neurological deficit and intracranial hypertension. Functional tumors secreting one or more hormones would result in some endocrinal symptoms. Non-functional tumors may affect the pituitary function and lead to hypopituitarism, like the first case. In our reported case, lesions invaded into the right cavernous sinus, suprasellar region, and nasal cavity leading to focal deficit such as bilateral exorbitism, left decreased vision, and CSF leak. On imaging, there is also no specificity. Bone erosion on CT scans, hypointensity on T1WI, hyperintensity on T2WI, and homogeneous enhancement are generous characteristics. Whole-body fluorodeoxyglucose-positron emission tomography (FDG-PET) would reveal primary lesions and metastasis. Recent researches suggested somatostatin receptors (SSTR) expressing on the surface of tumor cells could integrate with somatostatin analogue. According to this, SPECT scans with isotope-labeled somatostatin may be a possible way [5]. Biochemical measurement mainly focuses on hormone levels. Some literatures identified CgA that may become a tumor marker because it increased to 70–90 % among patients [6]. In the two present cases, the specimens are positive for CgA. Pathological diagnosis is the golden standard. The neoplasm is comprised of different size cells with uniform nuclei, scant cytoplasm, nidulant, and basophil granulocytes. Irregular mitosis presents based on the degree of differentiation [7]. CgA and Syn are selected as common immunohistochemical items sensitively and nonspecifically [8]. On the basis of recent literatures, peptide hormones and bioamine are no longer recommended to be tested immunohistochemically, because no correlation is confirmed between hormones and clinical presentations and the level of hormones also cannot give us valuable prognosis [9]. For the first case, immunohistochemical analysis of specimen was negative for all the hormones. On electron micrographs, intracytoplasmic neuroendocrine granules with compact cores show high electron density and have much value of differential diagnosis. For the second case, the tumor may have originated 6 years ago, but Cushing syndrome presented in only 2 months. Then, by following the postoperative condition for 1.5 years because of the residual fraction in nasal cavity, the patient has not presented hypercortisolism. The reason may be that NEGs were occasionally observed and not enough to cause the typical Cushing syndrome in a short period.

Diagnosis of NET depends on the pathological characteristics. Immunohistochemistry is one significant differential approach to neuroendocrine ingredients, epithelial ingredients, and neuropeptides. For NET in the sellar region, it should be necessary to discriminate it from esthesioneuroblastoma. Klimstra [7] thought esthesioneuroblastoma was comprised of neuroblasts with uniform circular nuclei and longitudinal cerebromedullary tubes in dendrites. NET comes from epithelium, lacking of neural ingredients. Depending on radiological findings of the second communicating case, most of the tumors situated intracranially in physiological canals in the skull base limited the tumor growth, resulting in minority extending outside the cranial via these canals.

#### Ectopic ACTH syndrome

Ectopic secretion of active ACTH or ACTH analogues from the non-pituitary tumors causes ectopic ACTH syndrome, resulting in the presences of Cushing syndrome [10]. Overt and occult lesions both account for ectopic secretion. However, the second immunohistochemistry was negative for ACTH. The main reasons may be as follows. (1) Ectopic tumors may excrete ACTH or ACTH analogue, and the simplex ACTH antibody is insufficient of covering the entirety. (2) Some lesions can produce ACTH and corticotrophin-releasing hormone (CRH) simultaneously [11]. Moreover, Mubarak et al. [6] reported three cases of ectopic CRH tumors with hypercortisolemia, and they also discussed that postoperative ACTH level immediately dropped to normality if the tumor produced ACTH, by contrast, it gradually decreased if the tumor produced CRH. This patient’s postoperative ACTH level was normal after 16 days. Imaging examinations, including CT, MRI, and FDG-PET, are used to identify the tumor sources, check the pituitary, and distinguish it from Cushing disease. It is also necessary to evaluate the biochemical indicator, including electrolytes, glucose, and hormones. Some special tests can be performed when diagnosis is difficult, for example, high-dose dexamethasone test, CRH stimulation test, and inferior petrosal sinus sampling (IPSS) [12].

The key point of diagnosis is how to differentiate between ectopic ACTH syndrome and Cushing disease. The ACTH level of overt ectopic lesion is distinctly higher than that of Cushing disease, but it is approximately the same as Cushing disease in occult lesion. In accordance with recent researches, the standard is that serum cortisol is shown to be 14 % higher than the base after CRH-releasing test is carried out 15 and 30 min later [13]. The other key point is to localize the tumor source. If sellar MRI shows normality, multisite radiological scans are considered, such as the head and neck, the chest, the abdomen, or the pelvis. We found no lesions in other parts of the second patient, and the endocrinal malfunction was related to the anterior skull base tumor.

#### Treatment

The optimal treatment of NET is surgical resection of hormone-secreting lesion. Peng et al. [14] reported a case of cervical lymph node metastasis after primary intracranial NET being removed 2 years later. If so, primary lesions resection and lymph nodes dissection are both needed. We chose single transsphenoid and subfrontal approaches for the two cases. Because the majority of the first lesion is located in the sellar and suprasellar regions, vascular supply was not relatively rich. For some bulky vessels showed on the second MRI and the main components of the second tumor located in the anterior cranial base, we chose subfrontal approach, and meanwhile, skull base reconstruction was performed during surgery.

Patients receiving comprehensive treatment, including extended resection of tumor and completion of chemo-radiotherapy, are expected to have longer tumor-free lives than those patients receiving single surgery [10, 15]. Poorly differentiated cells are sensitive to radiation. The first patient with high level of Ki-67 index died of extensive metastasis and had no chance to receive radiotherapy. The second NET with low proliferation was recommended to take a follow-up examination. SSTR2 and SSTR5 on the surface of NET cells can integrate with somatostatin analogue. Some doctors used isotope-labeled drugs, such as ^90^Y-DOTA-octreotide (OCT), ^111^In-DTPA-OCT, and ^131^I-DTPA-OCT, to complete the inner irradiation [5]. Because of epithelial origin, drugs, such as adriamycin, 5-fluorouracil, and cisplatin, are chosen for epithelial neoplasm. Few reports described primary intracranial NET, so chemotherapy still needs researches to demonstrate its therapeutic efficiency.

## Conclusions

The mechanism, diagnosis, and treatment of NET are still challenging. Surgical resection followed by radiotherapy had demonstrated an effective treatment, and chemotherapy still needs researches to demonstrate its therapeutic efficiency.

## Consent

Written informed consent was obtained from the first patient’s parents because of her death and the second patient herself for publication of this case report and any accompanying images. A copy of the written consent is available for review by the Editor-in-Chief of this journal. The ethical review has been approved by our ethics committee.

## Abbreviations

ACTH: adrenocorticotropic hormone
CgA: cytokeratin A
ChrA: chromogranin A
CRH: corticotropin-releasing hormone
CT: computed tomography
DOTA: tetraazacyclododecane tetraacetic acid
EAS: ectopic ACTH syndrome
FBG: fasting blood glucose
MRI: magnetic resonance imaging
NET: neuroendocrine tumor
OCT: octreotide
Syn: synaptophysin
